# A mini-review: environmental and metabolic factors affecting aminoglycoside efficacy

**DOI:** 10.1007/s11274-022-03445-8

**Published:** 2022-11-09

**Authors:** Calum M. Webster, Mark Shepherd

**Affiliations:** School of Biosciences, RAPID Group, University of Kent, Canterbury, CT2 7NJ UK

**Keywords:** Aminoglycoside, Proton motive force, Reactive oxygen species

## Abstract

Following the discovery of streptomycin from *Streptomyces griseus* in the 1940s by Selman Waksman and colleagues, aminoglycosides were first used to treat tuberculosis and then numerous derivatives have since been used to combat a wide variety of bacterial infections. These bactericidal antibiotics were used as first-line treatments for several decades but were largely replaced by ß-lactams and fluoroquinolones in the 1980s, although widespread emergence of antibiotic-resistance has led to renewed interest in aminoglycosides. The primary site of action for aminoglycosides is the 30 S ribosomal subunit where they disrupt protein translation, which contributes to widespread cellular damage through a number of secondary effects including rapid uptake of aminoglycosides via elevated proton-motive force (PMF), membrane damage and breakdown, oxidative stress, and hyperpolarisation of the membrane. Several factors associated with aminoglycoside entry have been shown to impact upon bacterial killing, and more recent work has revealed a complex relationship between metabolic states and the efficacy of different aminoglycosides. Hence, it is imperative to consider the environmental conditions and bacterial physiology and how this can impact upon aminoglycoside entry and potency. This mini-review seeks to discuss recent advances in this area and how this might affect the future use of aminoglycosides.

## Introduction

Aminoglycosides were first discovered as a natural product class of antibiotics in the 1940s (Becker and Cooper 2013), starting with Waksman, Schatz, and Bugie isolating streptomycin from *Streptomyces griseus* in 1944 (Schatz et al. 1944). Streptomycin was the first antibiotic treatment for tuberculosis (Daniel 2006), and the aminoglycosides are now broad-spectrum bactericidal antibiotics used mainly to treat aerobic Gram-negative bacteria and selected Gram-positive bacteria often in combination with other antibiotics (see review articles: Avent et al. 2011; Jackson et al. 2013; Fosso et al. 2014). The first aminoglycosides discovered were isolated from species of the genera *Streptomyces* or *Micromonospora* (Jana and Deb 2006). Aminoglycosides derived from *Streptomyces* (suffix -mycin) include kanamycin, apramycin, neomycin and tobramycin (Edson and Terrell 1987), whereas those produced by *Micromonospora* (suffix -micin) include gentamicin (Weinstein 1963) and sisomicin (Miller et al. 1976). Semisynthetic derivatives have since been developed to improve the pharmacological profile (Tod et al. 2000) and combat antimicrobial resistance (Krause et al. 2016), and examples include amikacin (Kawaguchi et al. 1972), netilmicin (Miller et al. 1976), isepamicin (Nagabhushan et al. 1978), arbekacin (Kondo et al. 1973), etimicin (Fan et al. 1995), and plazomicin (Aggen et al. 2010). Aminoglycosides entered widespread clinical use to combat infections caused by members of the Enterobacterales order of Gram-negatives including *Escherichia coli* and *Klebsiella pneumonia* (Krause et al. 2016), and they have also been used effectively against *Pseudomonas aeruginosa* (Karlowsky et al. 2003) and *Staphylococcus aureus* (Lee and Lee 2016). Alternative broad-spectrum antibiotics were developed in the 1970 and 1980 s that exhibited reduced toxicity compared to aminoglycosides (Becker and Cooper 2013), including fluoroquinolones, β-lactams combined with β-lactamase inhibitors, cephalosporins and carbapenems. This resulted in a decline in the development and use of aminoglycosides (Böttger and Crich 2020), although the increasing emergence of antimicrobial resistance has led to renewed interest in aminoglycosides (Böttger and Crich 2020).

## Mechanism of action

The primary target site of aminoglycosides is the bacterial ribosome, more specifically the 30 S subunit (Carter et al. 2000). A number of X-ray and NMR structures have been solved for different aminoglycosides binding to the ribosomal 30 S subunit, including well-known NMR structures of the neomycin class of aminoglycosides binding to the aminoacyl-tRNA recognition site (A-site) and interacting with the 16 S ribosomal RNA (Fourmy et al. 1998). X-ray crystallography later confirmed that streptomycin, spectinomycin and paromomycin also interact with the 16 S rRNA at the A-site (Carter et al. 2000). Binding of aminoglycosides to the A-site leads to the inhibition of protein synthesis as well as single/multiple misreading errors in translation(Parajuli et al. 2021) that can lead to amino acid substitutions and/or the production of truncated proteins (Davis 1987; Wohlgemuth et al. 2021).

**Fig. 1:**
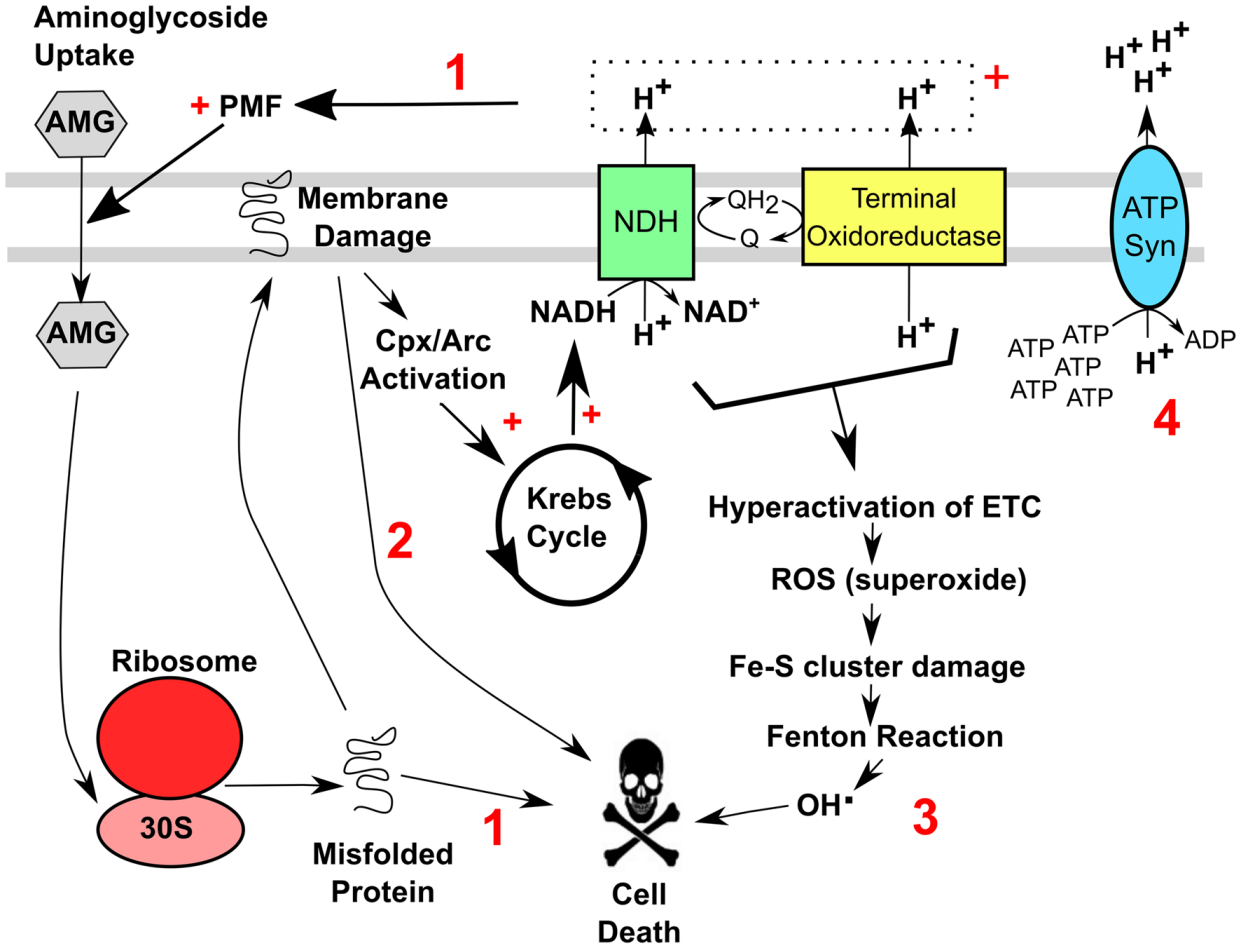
Current dogma on cell death pathways for aminoglycosides. The main routes for aminoglycoside bactericidal action are: (1) continuous uptake of aminoglycosides from positive feedback derived from an increase in the aerobic respiratory rate and PMF resulting in complete ribosomal inhibition; (2) insertion of mistranslated/misfolded proteins into the plasma membrane leading to membrane damage and breakdown; (3) oxidative stress from respiration-derived reactive oxygen species (ROS); (4) Hyperpolarisation of the membrane by the ATP synthase working in reverse. Abbreviations: AMG, aminoglycoside; ETC, electron transport chain; PMF, proton-motive force; NDH, NADH dehydrogenase; Q, ubiquinone; QH_2_, ubiquinol, *ROS* reactive oxygen species

Precisely how protein synthesis errors lead to the bactericidal effects of aminoglycosides is not fully understood. The main hypotheses that have been proposed to explain cell death from aminoglycosides (Fig. 1) are: (1) continuous uptake of aminoglycosides from positive feedback resulting in complete ribosomal inhibition (Ezraty et al. 2013); (2) insertion of mistranslated proteins into the membrane leading to membrane destabilisation and breakdown (Davis et al. 1986); (3) oxidative stress from reactive oxygen species (ROS) (Kohanski et al. 2007, 2008; Dwyer et al. 2015); (4) Dysregulation of membrane potential (Bruni and Kralj 2020). However, there is continuing debate as to which mechanism is the main route of cell death from aminoglycosides (Ezraty et al. 2013; Bruni and Kralj 2020).

## Aminoglycoside entry and bactericidal activity

In order for aminoglycosides to bind to bacterial ribosomes and exert bactericidal effects upon Gram-negative bacteria they must first cross both the outer and inner membranes. The most commonly cited model for aminoglycoside uptake comprises three stages (Taber et al. 1987). Firstly, an interaction occurs between anionic sites on the cell surface and the polycationic aminoglycosides (Hancock 1981; Vanhoof et al. 1995). Anionic binding sites include lipopolysaccharide (Vanhoof et al. 1995) and outer membrane proteins such as the porin OmpF in *E. coli* (Taber et al. 1987; Hancock et al. 1991). Membrane disruption then follows, allowing aminoglycosides into the periplasm (Hancock 1981). This first stage of uptake is not energy dependent, but this is followed by two energy dependent steps (EDP I/II) to cross the plasma membrane (Taber et al. 1987). EDPI is a slow uptake of aminoglycosides occurring immediately after exposure (Taber et al. 1987), and is a concentration-dependent process that occurs in both aminoglycoside-resistant and -sensitive bacterial cells (Taber et al. 1987). EDPII is a rapid energy-dependent uptake of aminoglycosides that proceeds via a mechanism that is poorly understood, although it is hypothesised to be vital for aminoglycosides to induce cell death (Taber et al. 1987).

The uptake of aminoglycosides is directly related to bacterial respiration, where the electrical component of the proton motive force (Δψ) is thought to provide the main driving force for the uptake of polar aminoglycosides (Taber et al. 1987; Farha et al. 2013). Indeed, early observations with species of *Clostridium* and *Bacillus* showed that sensitivity to aminoglycosides was strongly dependent upon an ability to perform nitrate- or oxygen-dependent electron transport, and careful control experiments were consistent with the hypothesis that the intrinsic resistance of *C. perfringens* to aminoglycosides is due to a lack of respiration-dependent uptake (Bryan et al. 1979).

Several uncouplers that dissipate the PMF have been used to study aminoglycoside uptake including 2,4-dinitrophenol (DNP) (Campbell et al. 1980) and carbonyl cyanide m-chloro phenylhydrazone (CCCP) (Miller et al. 1980). These uncouplers provide a pathway for protons to transport across the inner membrane leading to uncoupling of the PMF (Lewis et al. 1994). Dissipation of the Δψ component of the PMF using uncouplers has been shown to reduce aminoglycoside uptake and susceptibility (Campbell et al. 1980). This has been demonstrated for DNP in *E. coli* K-12 using dihydrostreptomycin (Campbell et al. 1980) and pathogenic *E. coli* EC958 using gentamicin (Webster et al. 2022). Ability to maintain the PMF by respiratory activity clearly contributes to the uptake of aminoglycosides and resultant bactericidal action.

External pH is known to influence the electrical component (ΔΨ) of the PMF, so it is logical to assume that pH may influence the uptake of aminoglycosides. Indeed, aminoglycoside activity is significantly diminished under low pH (Bryant et al. 1992; Hancock et al. 1981), and early work showed that increasing the pH from 6.0 to 9.0 enhances bactericidal activity of streptomycin (Donovick et al. 1948): while increasing pH up to 7.5 may enhance the electrical component (ΔΨ) of the PMF and potentially enhance uptake of aminoglycosides, ΔΨ does not change much above this pH (Felle et al. 1980) and increases in streptomycin efficacy above pH 7 are therefore more likely to result from enhancing the interactions between outer membrane anionic groups and cationic aminoglycosides (Hancock et al. 1981). On a related note, the presence of cations, particularly divalent cations, diminish aminoglycoside entry and efficacy, which is attributed to a competition with aminoglycosides for the anionic groups on the cell surface (reviewed in Hancock et al. 1981).

Once inside the cell, aminoglycoside-mediated bacterial killing is influenced via number of mechanisms (Fig. 1), and the toxic mechanisms of various antibiotic classes has been recently reviewed in (Baquero and Levin 2021). Mistranslated membrane proteins can be inserted into the membrane resulting in direct membrane damage (Davis et al. 1986; Kohanski et al. 2008) that results from the creation of irregular membrane channels leading to increased irreversible aminoglycoside uptake and eventual complete ribosome inhibition and cell death (Davis et al. 1986). Evidence has also been reported for misfolded membrane proteins activating the Arc and Cpx stress responses systems that elicit perturbations in central carbon metabolism and respiratory activity (Kohanski et al. 2008; Dwyer et al. 2014). Hyperactivation of the electron transport chain is predicted (Kohanski et al. 2008) which in turn leads to oxidative stress and cell death by hydroxyl radicals (Kohanski et al. 2007, 2008). This is predicted to be via superoxide production leading to damaged Fe-S clusters, release of ferrous iron and production of hydroxy radicals from oxidation of ferrous iron in the Fenton reaction (Kohanski et al. 2007; Dwyer et al. 2015). The importance of ROS in aminoglycoside-mediated cell death has long been debated, with other studies suggesting that the ROS response is not required for cell death (Leviton et al. 1995; Ezraty et al. 2013). The respiratory chain is well-known to support the maintenance of the PMF, and aminoglycoside-mediated hyperactivation is thought to promote the uptake of positively charged aminoglycosides through maintaining Δψ, leading to ribosome inactivation and cell death (Leviton et al. 1995; Ezraty et al. 2013; Webster et al. 2022). Finally, recent studies have reported that aminoglycoside exposure causes ATP accumulation (via loss of ATP consumption during protein synthesis) resulting in the export of protons by the ATP synthase working in reverse, which culminates in hyperpolarisation of the membrane. Hence, the literature supports the hypothesis that respiratory activity potentiates aminoglycoside-mediated cell death via a combination of ROS formation and the ability to maintain a PMF, and that this antibiotic class can elicit membrane hyperpolarisation that directly causes cell death.

## Decrease of aminoglycoside efficacy by nitric oxide-mediated respiratory inhibition

Environmental conditions that affect bacterial respiration have been reported to result in changes to aminoglycoside susceptibility and uptake, with nitric oxide (NO) serving as a well-known example that is important for aminoglycoside function during infection as well as in bacterial isolates found in the soil. Bacterial nitric oxide synthase (bNOS) enzymes exist in Gram-positive bacteria and loss of bNOS has previously been shown to diminish the ability of *Bacillus subtilis* to grow in the presence of aminoglycosides (Gusarov et al. 2009), which supports the hypothesis that endogenously-generated NO can enable bacteria to live in mixed microbial communities with aminoglycoside-producing microorganisms. Furthermore, NO can be produced by the human immune system as a response to infection (Espey et al. 2000; Wink et al. 2011) and can therefore be found in the host microenvironment, and exogenously delivered NO has been shown to decrease the lethality of aminoglycosides towards *E. coli* (Zhang et al. 2020; Ribeiro et al. 2021), *P. aeruginosa*, *S. aureus* and *Salmonella* (McCollister et al. 2011). In *E. coli*, NO has been shown to diminish the proton motive force (Webster et al. 2022), while in *Salmonella* a reduction in gentamicin uptake was observed in response to NO exposure (McCollister et al. 2011). NO is known to bind to the terminal respiratory oxidases in *E. coli* resulting in inhibition, especially towards the heme-copper oxidase cytochrome *bo′* (Mason et al. 2009; Zhang et al. 2020). It is therefore proposed that NO reduces the PMF by inhibiting the terminal cytochrome oxidases leading to the blocking of the EDP I and EDP II phases of aminoglycoside uptake (McCollister et al. 2011; Zhang et al. 2020). This is interesting as exogenous NO appears to be an attractive new antimicrobial (Schairer et al. 2012; Chiarelli et al. 2021; Michaelsen et al. 2021) with potential for combination approaches with cytochrome *bc*_1_ inhibitors (Zeng et al. 2021), although the literature resoundingly indicates that NO diminishes the efficacy of aminoglycosides and therefore a combination approach with NO would be ill advised.

## Elevation of aminoglycoside uptake and bactericidal activity by metabolites

**Fig. 2:**
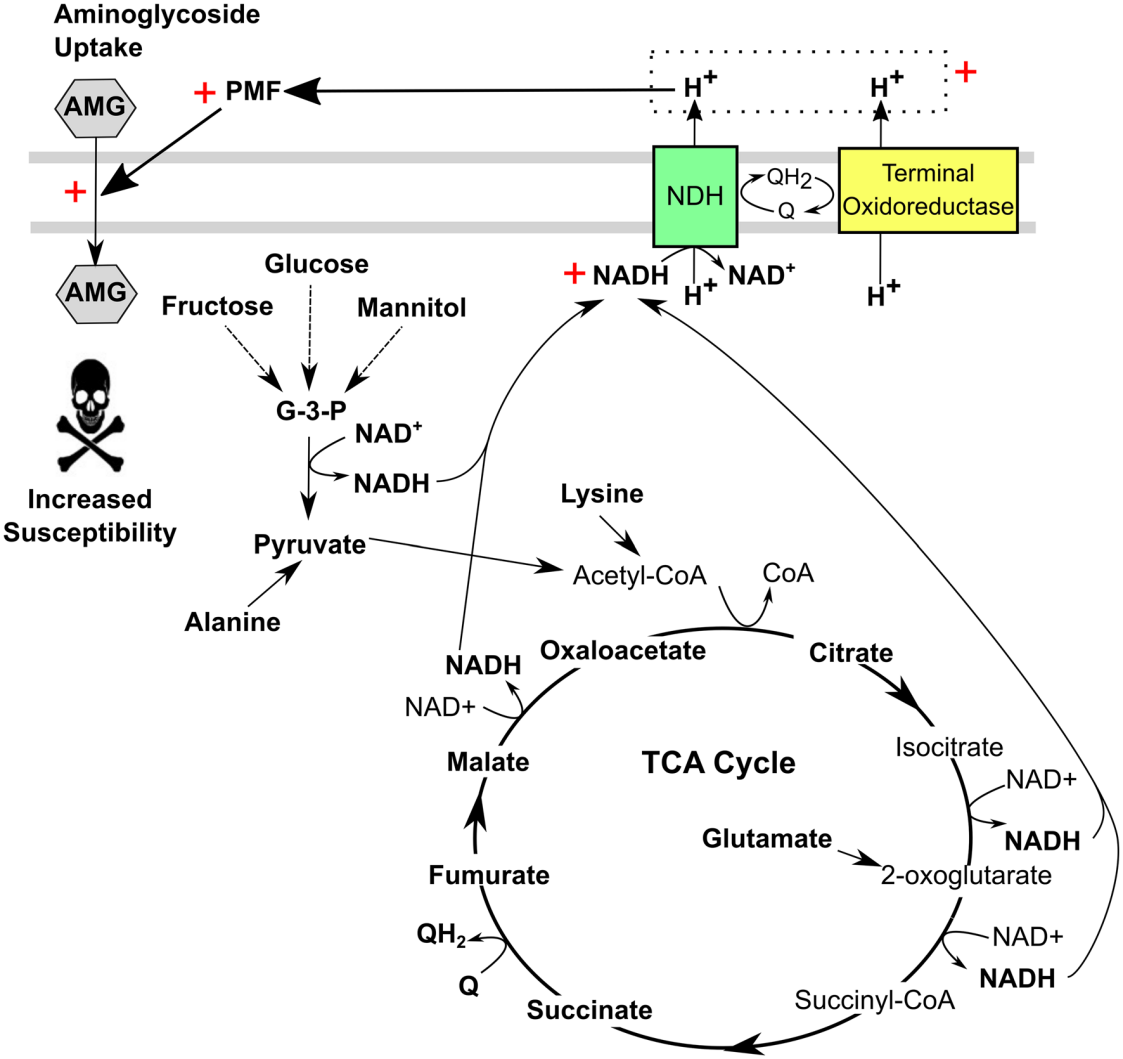
Overview of the PMF-dependent potentiating effect of metabolites on aminoglycoside susceptibility in bacteria. Highlighted metabolites in bold have been demonstrated to increase susceptibility to aminoglycosides via increased drug uptake. This is hypothesised to be via increasing flux through central carbon metabolism leading to increased NADH production, increased respiration rate and a consequent increase in the PMF. Aminoglycoside uptake has been shown to be directly related to the electrical potential component of the PMF. *AMG* aminoglycoside; G-3-P, glyceraldehyde-3-phosphate; *PMF* proton-motive force; *NDH,* NADH dehydrogenase; Q, ubiquinone; QH_2_, ubiquinol

**Fig. 3:**
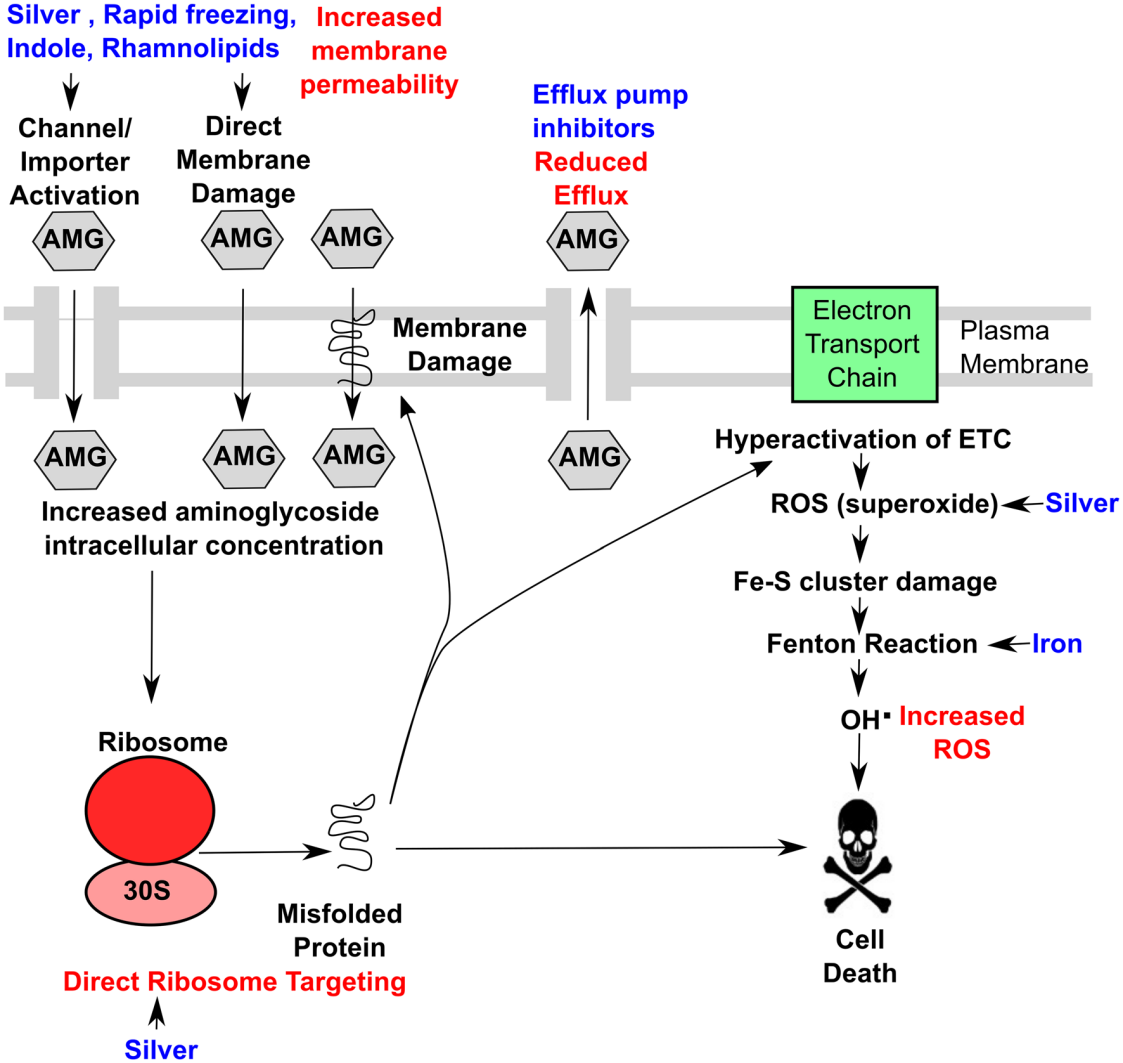
PMF-independent routes for increasing bactericidal activity of aminoglycosides. External effectors are in blue, mechanisms are in red, and cellular consequences are in black. *AMG,* aminoglycoside; *ETC,* electron transport chain; *PMF,* proton-motive force; Q, *ROS* reactive oxygen species

A variety of metabolites and environmental conditions have previously been shown to increase aminoglycoside bactericidal activity (Tables 1 and 2). Stimulation of central carbon metabolism via supplementation with carbon sources, metabolites and tricarboxylic acid (TCA) cycle components has been shown to elevate aminoglycoside uptake and susceptibility in a variety of bacterial species in a PMF-dependent manner (Table 1; Fig. 2) (Martins and Nguyen 2017). These include alanine (Kuang et al. 2021), glucose (Peng et al. 2015), glycine (Su et al. 2018), fumarate (Martins and Nguyen 2017; Hall et al. 2019), glyoxylate (Martins and Nguyen 2017), L-lysine (Deng et al. 2020), succinate (Crabbé et al. 2019), pyruvate (Crabbé et al. 2019), glutamate (Su et al. 2018) and citrate (Su et al. 2018). Other metabolites such as indoles (Sun et al. 2020) show adjuvant activity with aminoglycosides in a manner independent of the PMF or act via several routes (Fig. 3; Table 1). These studies have generally focused on aminoglycoside susceptibility in aerobic Gram-negative bacteria including pathogenic strains of *E. coli, A. baumannii, K. pneumoniae* and *P. aeruginosa*, although studies on the Gram-positive methicillin resistant *S. aureus* (MRSA) are also reported.

**Table 1:** Metabolite stimulators of bactericidal activity of aminoglycosides

Metabolite	Hypothesised mechanism	Bacterial species	References
Glucose	PMF dependent	*K. pneumoniae, P. aeruginosa, S. aureus*	Peng et al. (2015)
Mannitol	PMF dependent	*E. coli*	Allison et al. (2011a)
Fructose	PMF dependent	*E. coli, S. aureus*	Allison et al. (2011a)
Pyruvate	PMF dependent	*E. coli, E. tarda*	Su et al. (2018)
Succinate	PMF dependent	*P. aeruginosa*	Crabbé et al. (2019)
Citrate	PMF dependent	*E. coli, E. tarda*	Su et al. (2018)
Oxaloacetate	PMF dependent	*E. coli, E. tarda*	Su et al. (2018)
Fumarate	PMF dependent	*P. aeruginosa*	Meylan et al. (2017)
Alanine	PMF dependent	*V. alginolyticus, E. tarda, K. pneumonia, P. aeruginosa, S. aureus*	Peng et al. (2015); Kuang et al. (2021)
Lysine	PMF dependent	*E. coli, A. baumannii, K. pneumoniae, M. smegmatis*.	Deng et al. (2020)
Glutamate	PMF dependent	*E. coli, E. tarda, P. aeruginosa*	Su et al. (2018); Crabbé et al. (2019)
5-methyl indole	PMF independent	*S. aureus, S. pyogenes, E. faecalis, S. epidermidis, Lysobacter *spp*.*	Sun et al. 2020) Wang et al. (2019)

The hypothesised mechanism of aminoglycoside potentiation is displayed along with bacterial species tested

Amino acids such as alanine and L-lysine have been shown to increase the toxicity of aminoglycosides (Peng et al. 2015; Deng et al. 2020). In the case of L-lysine, an increase in the PMF has been recorded in the Gram-negative pathogen *Acinetobacter baumannii* along with an increase in aminoglycoside potency leading to the conclusion that this is due to increased uptake of aminoglycosides (Deng et al. 2020). Interestingly, an increase in intracellular ROS was also observed with exposure to both L-lysine and aminoglycosides compared to exposure to aminoglycoside or L-lysine individually (Deng et al. 2020). Multiple studies in a variety of Gram-negative pathogenic bacteria, including *Edwardsiella tarda* (Peng et al. 2015; Su et al. 2018), *Vibrio alginolyticus* (Jiang et al. 2020; Kuang et al. 2021)d *coli* (Jiang et al. 2020), have demonstrated that alanine elevates aminoglycoside potency. The proposed mechanism of action starts with alanine feeding into the TCA cycle via pyruvate leading to increased metabolic flux through the TCA cycle, increased production of NADH, enhanced respiration rates, increased PMF and finally increased uptake of aminoglycosides (Fig. 2) (Peng et al. 2015). Exogenous addition of alanine and glucose has also been shown to restore kanamycin sensitivity to multidrug-resistant *E. tarda* (Peng et al. 2015).

Exposure to alanine resulted in an enhancement of susceptibility to gentamicin in the pathogen *Vibrio alginolyticus* (Kuang et al. 2021). This effect was shown to be mediated by alanine that reduced the production of NO by CysJ, which uses nitrite as a substrate (Kuang et al. 2021). Metabolomic analysis revealed fluctuations in the TCA cycle and the urea cycle with the latter involved in NO production (Kuang et al. 2021). The decrease in NO levels was proposed to be the key factor for the increased susceptibility to gentamicin when combined with alanine in *Vibrio alginolyticus* (Kuang et al. 2021).

Various metabolites have been explored as adjuvants to sensitise bacterial persister cells to aminoglycosides (Allison et al. 2011a). Bacterial persisters occur where a sub-population of cells survive antibiotic treatment and are often found in biofilm infections where the cells are dormant in stationary phase (Allison et al. 2011b). Metabolites involved in sugar catabolism including glucose, fructose, mannitol and pyruvate, have been shown to induce gentamicin-mediated killing of *E. coli* persisters in biofilms (Allison et al. 2011a). These metabolites were also shown to increase the PMF in the *E. coli* cells (Allison et al. 2011a), which supports the hypothesis that stimulation of central carbon metabolism increases respiratory rates and subsequent PMF generation that leads to increased uptake of aminoglycosides and cell death (Fig. 2) (Allison et al. 2011a). Fructose was also shown to potentiate aminoglycoside efficacy against *S. aureus* persister cells, although glucose, mannitol and pyruvate had no effect (Allison et al. 2011a). The C_4_-dicarboxylates such as succinate and fumarate involved in the TCA cycle are also linked to aminoglycoside susceptibility (Martins and Nguyen 2017; Meylan et al. 2017). Addition of fumarate to starved tobramycin-tolerant *P. aeruginosa* was shown to sensitize the cells to tobramycin-mediated killing (Meylan et al. 2017). This suggested that addition of fumarate stimulated TCA cycle activity which in turn elevated respiration in the stationary phase starved cells, leading to increased PMF and a subsequent increase in tobramycin uptake (Fig. 2) (Meylan et al. 2017). This process is dependent on tobramycin transporters regulated by the alternative sigma factor RpoN (Hall et al. 2019). Furthermore, addition of metabolites to the growth medium that feed into the TCA cycle such as glucose, pyruvate and acetate, also results in increased killing of *P. aeruginosa* by tobramycin (Meylan et al. 2017). Interestingly, glyoxylate diminished tobramycin-mediated killing through increasing the glyoxylate shunt resulting in lower NADH and FADH_2_ levels that decreased respiratory activity (Martins and Nguyen 2017; Meylan et al. 2017). These studies demonstrate the importance of bacterial carbon catabolism and respiration in the modification of aminoglycoside susceptibility related to the uptake of aminoglycosides. This highlights the potential for using PMF-stimulating metabolites as aminoglycoside adjuvants to treat infections.

The potentiating effect of indoles upon aminoglycoside action has been investigated in a number of studies with some reporting increased susceptibility (Kwan et al. 2015; Wang et al. 2019; Sun et al. 2020) whilst others reporting decreased aminoglycoside susceptibility (Han et al. 2011; Vega et al. 2013). One of the latest studies found that 5-methyl indole potentiated tobramycin activity against Gram-positive MRSA persister cells along with other Gram-positives but not against Gram-negative *E. coli* and *Shigella flexneri* (Sun et al. 2020). Interestingly, treatment with the PMF-disrupting protonophore CCCP resulted in little effect on the potentiation of tobramycin activity by 5-methyl indole, suggesting a PMF-independent mechanism for aminoglycoside potentiation (Fig. 3) (Sun et al. 2020). The mechanism for the potentiating effect of indoles is unclear and may be unique to Gram-positives and possibly involve membrane disruption (Sun et al. 2020) or cell signalling (Vega et al. 2013; Kwan et al. 2015; Wang et al. 2019). The effect of indoles upon the bactericidal action of aminoglycoside are likely to be strain-dependent and also related to the experimental conditions used, as addition of salts including MgCl_2_ and CaCl_2_ has been shown to abolish the potentiating effects of indoles and aminoglycosides in MRSA (Sun et al. 2020).

In vivo studies involving aminoglycoside susceptibility are far less common than in vitro assays. One study looked at the host metabolomic effects in a 3D lung cell model (Crabbé et al. 2019). Interestingly, tobramycin uptake and bactericidal action was increased in a range of *P. aeruginosa* strains when grown within the 3D lung model (Crabbé et al. 2019). The PMF was also increased in *P. aeruginosa* when in the 3D model compared to the 2D monolayer cultures (Crabbé et al. 2019). In the 3D lung cell model, secreted host metabolites such as succinate and glutamate were proposed to be responsible for the increased tobramycin uptake due to increased PMF resulting from stimulation of the TCA cycle and increased respiration (Crabbé et al. 2019). This work highlights the importance of considering the host microenvironment when considering aminoglycoside uptake and susceptibility. Another in vivo study investigated the synergy between gentamicin and mannitol in *E. coli* biofilms in catheters inserted into the urinary tract of mice (Allison et al. 2011a). Gentamicin on its own had zero effect upon biofilm viability but when combined with mannitol the viability was reduced by around 1.5 orders of magnitude (Allison et al. 2011a). A further study showed that the indole 5-methyl indole displays tobramycin potentiation against Gram-positive MRSA in a mouse skin cell model (Sun et al. 2020).

## Elevation of aminoglycoside uptake and bactericidal activity by non-metabolites and physical treatments

Various substances and physical treatments have been shown to modify aminoglycoside susceptibility, with some acting via a PMF-dependent route whilst others were shown to act independently of the PMF (Fig. 3) (Table 2). The first example to be address herein is butanol, which is well-known to impair membrane integrity and can induce leakiness (Fletcher et al. 2016) that could potentially facilitate antibiotic uptake. Indeed *n*-butanol has been investigated as an aminoglycoside potentiator against various antibiotic tolerant persisters including Gram-positive MRSA (Lv et al. 2022). Whilst not being a metabolite of MRSA, butanol was seen to enhance the uptake of the aminoglycoside tobramycin in a PMF-dependent manner leading to increased killing of this bacterium (Lv et al. 2022).

In addition to xenobiotic organic compounds such as butanol, inorganic iron has also been investigated as an adjuvant for aminoglycosides. Reviewed in (Ezraty and Barras 2016), iron has been shown to both decrease or increase aminoglycoside susceptibility depending on the bacterial strain and assay conditions used. For example, decreasing the iron level was shown to increase aminoglycoside resistance in *E. coli* but decrease resistance in *K. pneumoniae* (Ezraty and Barras 2016). Iron can play a role in ROS production via the Fenton reaction (Kohanski et al. 2007; Dwyer et al. 2015) leading to cell damage and has also been implicated in aminoglycoside uptake via maintenance of the PMF (Ezraty et al. 2013). It is thought that high iron levels promote expression of the Isc machinery that enhances Fe-S cluster assembly in succinate dehydrogenase and NADH dehydrogenase in *E. coli*, which facilitates respiration and maintenance of the PMF for aminoglycoside uptake (Ezraty et al. 2013; Ezraty and Barras 2016). In addition, multiple two component signalling systems and transcription factors can interact with iron, leading to changes in cell membrane permeability or drug efflux (Ezraty and Barras 2016) resulting in a complicated picture regarding iron and aminoglycosides (Fig. 3).

Silver ions also have antimicrobial properties and are known to target DNA, Fe-S proteins and bacterial membranes (Barras et al. 2018). Silver has been used in conjunction with a number of antibiotic classes as an adjuvant (Barras et al. 2018). Silver has also been used to successfully potentiate aminoglycoside bactericidal activity against *E. coli* and to sensitise a gentamicin resistant strain (Morones-Ramirez et al. 2013; Herisse et al. 2017). Furthermore, silver was also shown to sensitise the aminoglycoside resistant anaerobic pathogen *Clostridium difficile* to gentamicin, tobramycin and kanamycin (Herisse et al. 2017). The mechanism for silver enhancing aminoglycoside action is a little contentious and has been reported to: (i) be PMF independent with silver-induced membrane permeabilisation promoting uptake; (ii) directly induce ROS formation: (iii) possibly directly target protein translation to exacerbate aminoglycoside-mediated ribosome dysfunction (Fig. 3) (Park et al. 2009; Morones-Ramirez et al. 2013; Herisse et al. 2017).

**Table 2:** Non-metabolite stimulators of bactericidal activity of aminoglycosides

Effector	Hypothesised mechanism	Bacterial species	References
*n*-butanol	PMF dependent	*S. aureus, S. pyogenes, E. faecalis, S. epidermidis*,	Lv et al. (2022)
Iron	PMF dependent and PMF independent	*E. coli*	Kohanski et al. (2007); Ezraty et al. (2013); Dwyer et al. (2015); Ezraty and Barras (2016)
Silver	PMF independent	*E. coli, C. difficile*	Morones-Ramirez et al. (2013); Herisse et al. (2017)
Rhamnolipids	PMF independent	*S. aureus*	Radlinski et al. (2019)
Rapid freezing	PMF independent	*E. coli, P. aeruginosa*	Zhao et al. (2020)

The hypothesised mechanism of aminoglycoside potentiation is displayed along with the bacterial species tested

Other non-metabolites studied as potential aminoglycoside adjuvants include antimicrobial lipids such as rhamnolipids that are produced by *P. aeruginosa* (Radlinski et al. 2017). In this study, rhamnolipids were shown to sensitise *S. aureus* persister cells to tobramycin and also sensitised *S. aureus* biofilm associated or anaerobic cultures to tobramycin killing. Rhamnolipids cause membrane damage to Gram-positive bacteria through a poorly understood mechanism that leads to increased small molecule permeability, and a PMF-independent increase in tobramycin uptake was observed on treatment with rhamnolipids (Fig. 3) (Radlinski et al. 2019). Antibacterial lipids such as rhamnolipids do have problems with cytotoxicity as they can also target eukaryotic cell membranes that will limit future treatment applications.

Efflux pump inhibitors have also been studied as potential adjuvants (Aygul 2015). Various molecules have been shown to inhibit efflux pumps including chlorpromazine, CCCP and reserpine (Aygul 2015; Sharma et al. 2019). However, there have been problems with efficacy and toxicity that have precluded clinical applications (Gupta and Datta 2019; Sharma et al. 2019). Also, some of these inhibitors such as CCCP work by reducing the PMF that many efflux pumps require to export aminoglycosides (Sharma et al. 2019). Reduction of the PMF will also reduce aminoglycoside import (Taber et al. 1987) and is also likely to lead to toxicity problems with mammalian cells, as is the case for CCCP (Sharma et al. 2019). Antibiotic hybrids are seen as more promising way of inhibiting efflux pumps (Gupta and Datta 2019). These are synthetic aminoglycoside hybrids that combine an aminoglycoside with another molecule that inhibits efflux: reviewed in (Gupta and Datta 2019).

Some physical treatments have also been shown to increase aminoglycoside uptake and susceptibility. Physical treatments such as rapid freezing in liquid nitrogen have been shown to increase aminoglycoside uptake by a PMF independent route through membrane disruption in *E. coli* and *P. aeruginosa* antibiotic tolerant persister cells (Zhao et al. 2020). Although successful in a mouse skin wound model (Zhao et al. 2020) there is the obvious application problems of damage to host cells caused by the freezing process, combined with the inability to treat systemic infections.

## Membrane potential dysregulation potentiates the bactericidal activity of aminoglycosides

**Fig. 4:**
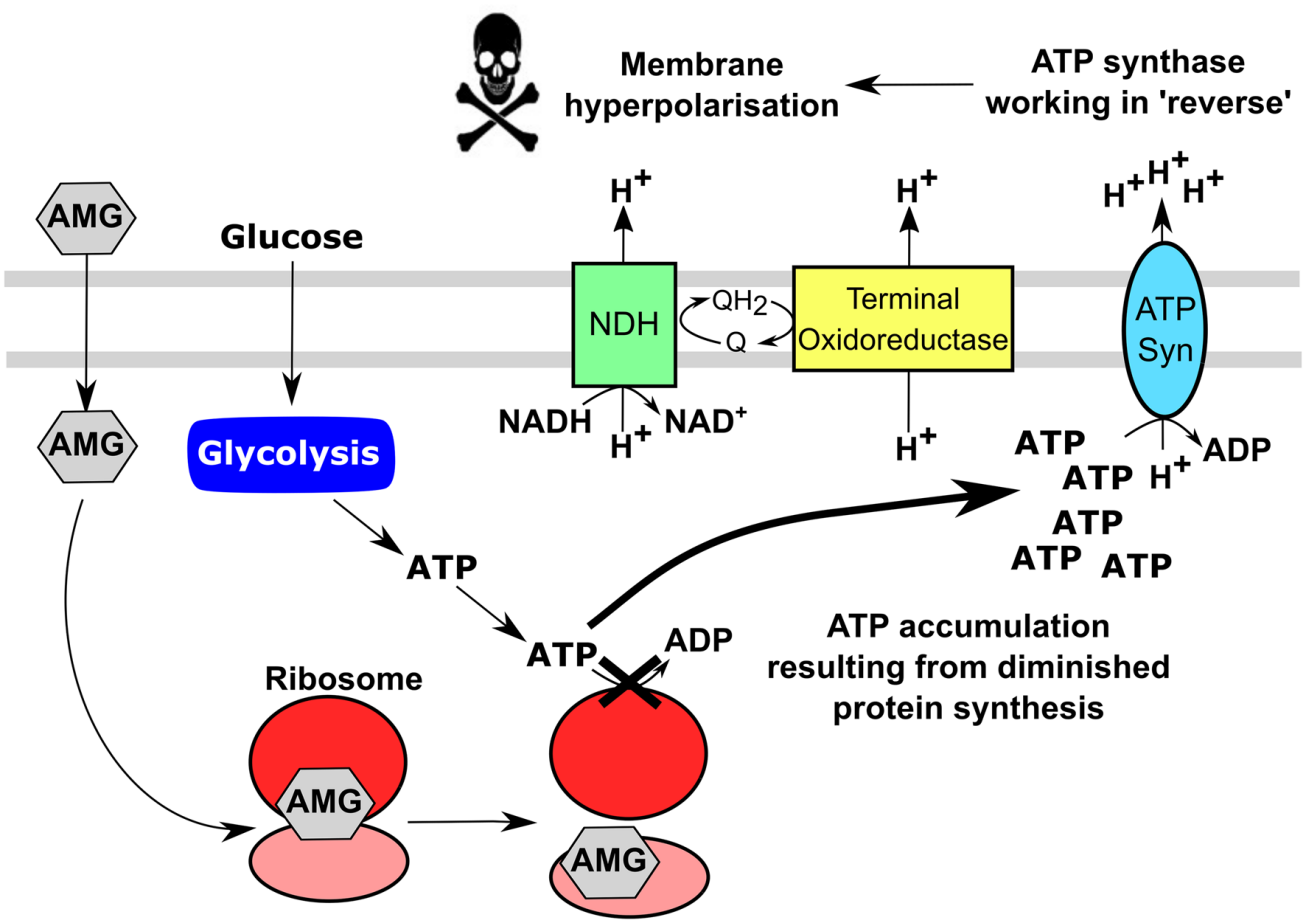
Aminoglycoside-mediated ATP accumulation results in membrane hyperpolarisation. The loss of ATP consumption that results from diminished protein synthesis causes ATP accumulation and elicits hydrolysis and proton export by the ATP synthase. This results in bactericidal hyperpolarisation of the membrane. *AMG* aminoglycoside; *NDH,* NADH dehydrogenase; Q, ubiquinone; QH_2_, ubiquinol

Single cell fluorescence measurements have recently highlighted the impact that membrane potential has in response to inhibitors of protein translation (Lee et al. 2019). In this study Lee et al. used microfluidics and a fluorescent dye to demonstrate that exposure of *Bacillus subtilis* cells to spectinomycin and kanamycin resulted in hyperpolarisation, a phenomenon that predominates in slower-growing cells that are much more susceptible to aminoglycoside-mediated killing. Hence, a negative correlation between growth rate and antibiotic-induced hyperpolarisation (and cell death) was demonstrated, which deviated from established dogma that supports dormant cells (e.g. in biofilms) typically being more resistant to aminoglycosides. This correlation was attributed to actively-growing cells being more able to correct the hyperpolarisation through the influx of magnesium ions, and represented a new mechanism that favours the survival of metabolically *active* cells. A subsequent study reported that hyperpolarisation was necessary for the bactericidal action of aminoglycosides against *E. coli* and proposed a model for “voltage induced death” (Fig. 4), which is consistent with many of the earlier observed relationships between PMF and aminoglycoside-mediated bacterial killing.

## Conclusion

Environmental conditions encountered during infection are well-known to modulate resistance to aminoglycosides in a variety of susceptible bacterial pathogens. Changes in bacterial respiration and metabolism have been shown to affect aminoglycoside uptake and bactericidal activity, and as a result many studies have focussed on altering central carbon metabolism and respiration in pathogenic bacteria in an attempt to improve the efficacy of aminoglycosides. A large number of metabolites, non-metabolites and physical treatments have been shown to sensitize bacterial pathogens to aminoglycosides via either PMF-dependent or PMF-independent routes. This dependence upon central carbon metabolism and respiratory activity is also consistent with the recent model for hyperpolarisation (Bruni and Kralj 2020), which presents a compelling argument for membrane voltage dysregulation being a major cause of aminoglycoside-mediated bacterial killing. In future it would be interesting to investigate which metabolites provide the greatest adjuvant effect with a number of bacterial species under the same test conditions, and to analyse correlations with membrane polarisation.

Some potential adjuvants are also hypothesised to act via non-PMF dependent routes such as via direct membrane damage or increased ROS. These treatments may be especially useful to target pathogens that are growing anaerobically under the low oxygen conditions often found during infection for example in biofilms (Herisse et al. 2017; Radlinski et al. 2019). However, it is important to note that aminoglycosides also work under anaerobic conditions, which would preclude the ROS route as a mechanism of toxicity.

Respiratory inhibitors such as exogenous NO treatment have previously been suggested as new antibacterial treatments (Chiarelli et al. 2021; Michaelsen et al. 2021) particularly in combination with other antimicrobials (Zeng et al. 2021). However, NO as a bacterial terminal respiratory complex inhibitor (Mason et al. 2009; Zhang et al. 2020) has been shown to reduce the PMF, which in the particular case of aminoglycosides leads to decreased susceptibility due to reduced uptake (McCollister et al. 2011; Zeng et al. 2021; Webster et al. 2022). Hence, caution should be taken when considering respiratory inhibitors as antibiotic adjuvants particularly in the case of aminoglycosides.

Most of the studies considered in this review assessed aminoglycoside susceptibility to synergy treatments using microbial growth conditions that did not accurately reflect the environmental conditions found during infection, which can have a profound effect upon the efficacy of certain treatments (Crabbé et al. 2019). Much further work is needed in this area if any synergy treatments are to reach the clinic. In vivo efficacy, dosage, off target side effects, route of administration and toxicity all need to be considered. Some potential adjuvants such as rhamnolipids (Radlinski et al. 2019) or physical treatments such as rapid freezing (Zhao et al. 2020) can cause direct damage to human cells. Aminoglycosides can also induce side effects in host cells such as ototoxicity (Fu et al. 2021) which need to be considered when discussing potential adjuvants. There is also the possibility that some adjuvants may increase the off target toxic effects of aminoglycosides.

In summary, the use of adjuvants to increase aminoglycoside efficacy via inducing changes in the metabolic state of bacterial cells represents an exciting strategy to combat antimicrobial resistance and tolerance. This is particularly true for various pathogens such as *E. coli*, *K. pneumonia* and *P. aeruginosa*, and MRSA. Clearly, further work is needed in this area to determine the most effective combination treatments for individual bacterial species. Ideally, synergistic approaches should be broad acting, target a range of bacterial species and be effective for a number of different aminoglycosides. However, more in vivo studies are needed to assess adjuvant treatments under conditions found during infection, and considerations of off-target effects, administration routes, dosage, direct toxicity and indirect toxicity are needed before clinical applications can be considered.
